# 779. Make Micro Cool Again: Implementing a Longitudinal Lab-based Microbiology and Mycology Curriculum to Engage Infectious Diseases Fellows in Hands-On Learning

**DOI:** 10.1093/ofid/ofad500.840

**Published:** 2023-11-27

**Authors:** Erin Pollock, Vishakh C Keri, Hossein Salimnia, Lea M Monday

**Affiliations:** Wayne State University School of Medicine, Detroit, Michigan; Wayne State University, Detroit, Michigan; Wayne State University, Detroit, Michigan; Wayne state University School of Medicine, Detroit, Michigan

## Abstract

**Background:**

Microbiology wet labs have been eliminated in many medical schools and infectious diseases (ID) fellows are training in a post-COVID era where hands-on experience is lacking. The IDSA recommends 120 hours of microbiology experience for fellows, but feasibility is limited in centers struggling to retain adequate staff, and technicians are limited in their ability to integrate clinical skills. We aimed to overcome these struggles by implementing a microbiology and mycology curriculum for ID fellows using a case-based approach within the microbiology lab (Fig1).

**Figure:**
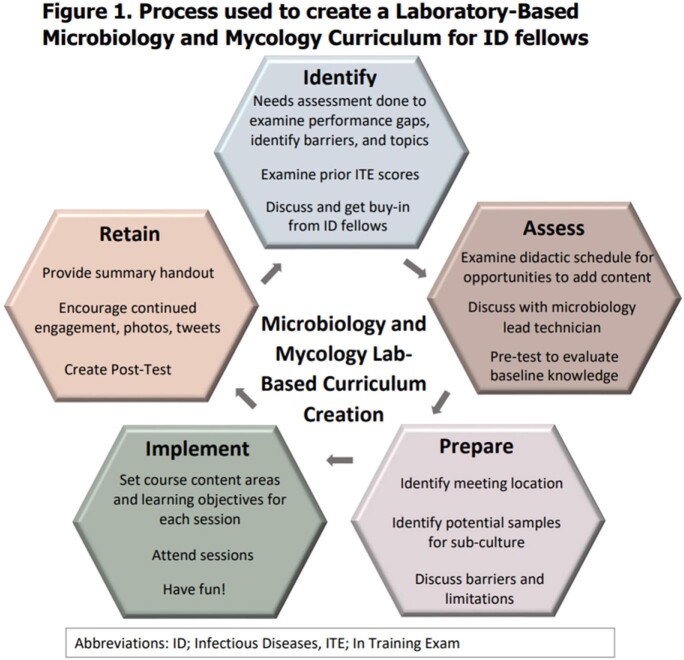

**Methods:**

In a quasi-experimental study, eight ID fellows from Wayne State University were taught in interactive, 1-hour sessions delivered monthly in the lab over 11 months using existing samples. Planning included a needs assessment, review of in training scores to identify weak spots, and pre-tests to assess baseline knowledge (Fig 1). Sessions topics are shown in Table 1. All sessions were delivered by an ID faculty to ensure homogeneity. Prior to each session, specimens were procured and objectives were outlined and linked to core content areas. An example of the objectives and core content for the hyaline mold session is shown in Table 2. A post-test occurred after session 9. Primary outcome was comparison of de-identified pre and post test scores by Wilcoxon signed-rank test for paired non-parametric data. Fellows experience and feedback for improvement were also collected.

**Figure:**
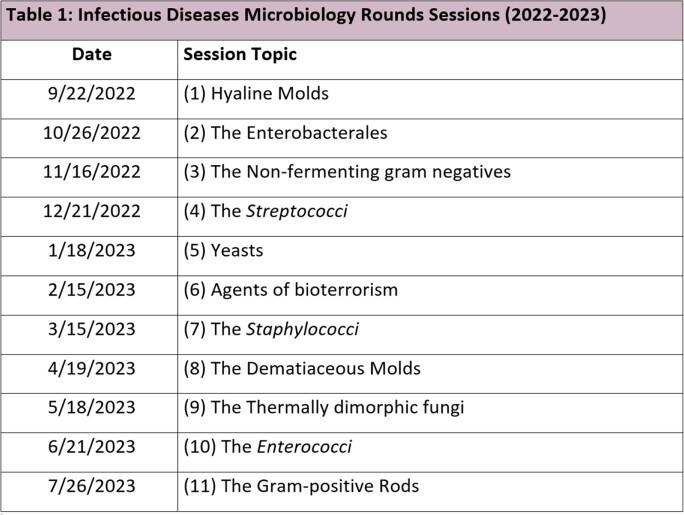

**Figure:**
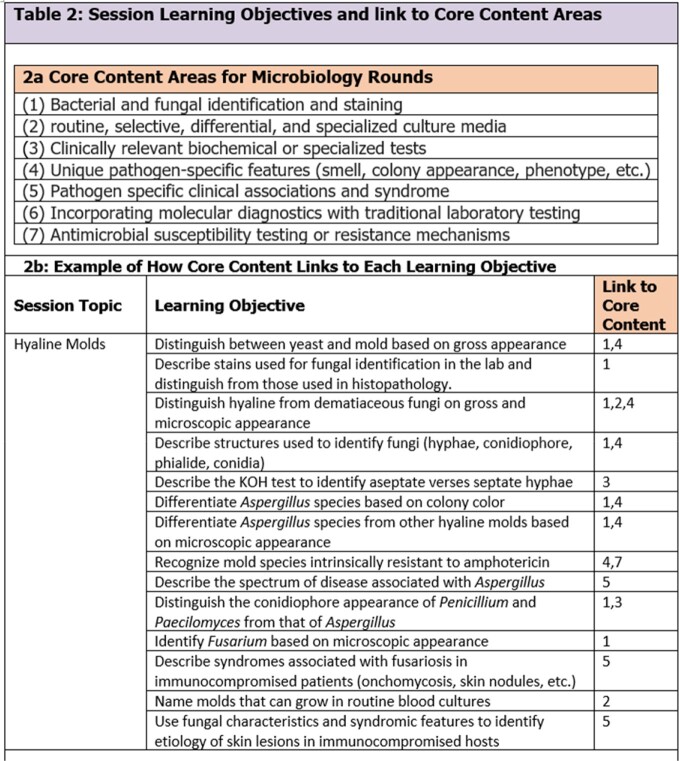

**Results:**

Sessions were performed as described and included hands on activities and a summary handout (Fig 2). Pre versus post test scores showed significant median improvement (65% to 82.5%. p=0.048, table 3). Feedback on the sessions was overwhelmingly positive, with suggestions for encore sessions and request to make a similar parasitology curriculum (Table 4). Sessions became so popular that pharmacists and other trainees began attending. Although not an intended outcome, it was noted that after posting handouts to twitter, fellowship following increased by 250%.

**Figure:**
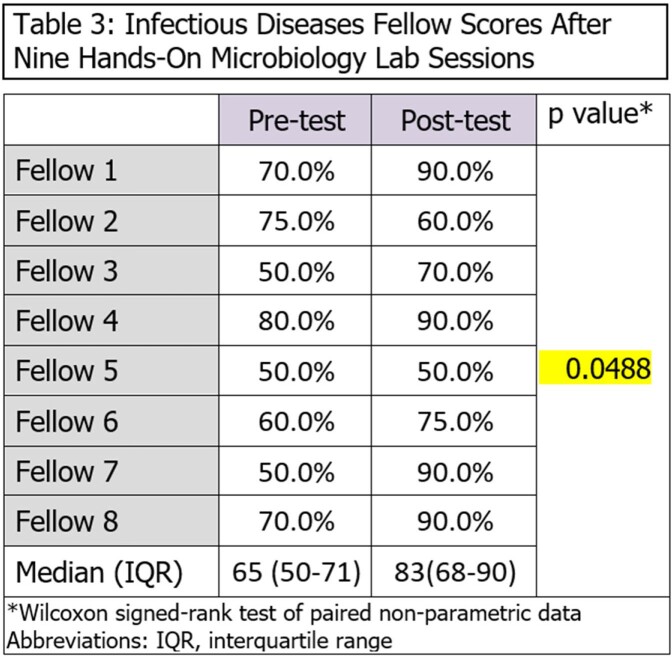

**Figure:**
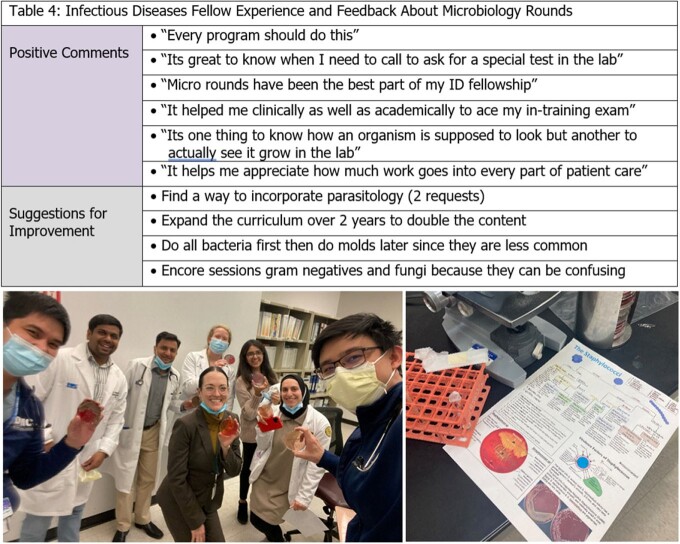

**Conclusion:**

A monthly longitudinal microbiology and mycology curriculum was well received by ID fellows and objectively increased their knowledge. This is the first study attempting to objectively quantify knowledge gained using a hands-on microbiology lab curriculum to teach ID fellows.

**Disclosures:**

**All Authors**: No reported disclosures

